# Notch-Mediated Suppression of TSC2 Expression Regulates Cell Differentiation in the *Drosophila* Intestinal Stem Cell Lineage

**DOI:** 10.1371/journal.pgen.1003045

**Published:** 2012-11-08

**Authors:** Subir Kapuria, Jason Karpac, Benoit Biteau, DaeSung Hwangbo, Heinrich Jasper

**Affiliations:** 1 Department of Biology, University of Rochester, Rochester, New York, United States of America; 2 Buck Institute for Research on Aging, Novato, California, United States of America; University of California San Francisco, United States of America

## Abstract

Epithelial homeostasis in the posterior midgut of *Drosophila* is maintained by multipotent intestinal stem cells (ISCs). ISCs self-renew and produce enteroblasts (EBs) that differentiate into either enterocytes (ECs) or enteroendocrine cells (EEs) in response to differential Notch (N) activation. Various environmental and growth signals dynamically regulate ISC activity, but their integration with differentiation cues in the ISC lineage remains unclear. Here we identify Notch-mediated repression of Tuberous Sclerosis Complex 2 (TSC2) in EBs as a required step in the commitment of EBs into the EC fate. The TSC1/2 complex inhibits TOR signaling, acting as a tumor suppressor in vertebrates and regulating cell growth. We find that TSC2 is expressed highly in ISCs, where it maintains stem cell identity, and that N-mediated repression of TSC2 in EBs is required and sufficient to promote EC differentiation. Regulation of TSC/TOR activity by N signaling thus emerges as critical for maintenance and differentiation in somatic stem cell lineages.

## Author Summary

Stem cells maintain tissue homeostasis in metazoans. A productive model to study the regulation of stem cell function is the *Drosophila* posterior midgut. Notch (N) signaling controls intestinal stem cell (ISC) differentiation in this tissue, while ISC proliferation is regulated by growth factor signaling pathways, including Insulin/IGF signaling (IIS). In this study, we explore the interaction between growth signals and N signaling in the control of ISC proliferation and differentiation. We show that TOR signaling, which promotes growth and can be activated by the IIS pathway, is maintained in ISCs in an inactive state by high expression of the TOR inhibitor TSC2. TSC2 expression shelters ISCs from nutritional cues, ensuring their long-term maintenance. In response to N pathway activation in enteroblasts (EB), the ISC daughter cells, TSC2 is transcriptionally repressed and TOR is activated. We demonstrate that this negative interaction between N and TSC2 is required and sufficient for differentiation of EBs into enterocytes (ECs), the absorptive cells of the epithelium. Our findings establish a critical role for TSC in ISC maintenance and provide a mechanism by which N promotes differentiation into the EC fate. The human homologue of TSC2 is an important tumor suppressor, and our study provides new insight into how its regulation controls regenerative processes.

## Introduction

Regenerative processes in somatic tissues require coordinated regulation of stem cell proliferation and daughter cell differentiation to ensure long-term tissue homeostasis [1]–[3]. The *Drosophila* posterior midgut epithelium has emerged as an excellent model system to study this regulation [4]–[7]. It is maintained by Intestinal stem cells (ISCs) that divide to self-renew and produce enteroblasts (EB), which undergo differentiation to become either enterocytes (ECs) or enteroendocrine cells (EEs) [8]–[10].

Differentiation in the ISC lineage is controlled by Delta/Notch (Dl/N) signaling. ISCs express Dl and activate N in EBs, thus promoting differentiation into either EEs or ECs. The cell fate decision between ECs and EEs seems to be regulated by the intensity of the Dl signal, i.e. high levels of N activity in EBs result in EC differentiation, while moderate activation of N promotes EE differentiation [10], [11]. Dl-mediated N activation in EBs increases the activity of the Suppressor of Hairless (Su(H)) transcription factor, presumably by replacing the Hairless transcriptional repressor from Enhancer of Split (E(spl)) complex promoters with the Notch intracellular domain (N^ICD^) [12]. How this pathway coordinates cell specification with cell growth and proliferation in the ISC lineage remains unclear.

ISC proliferation is regulated by growth factor and stress signaling pathways [13]–[32]. These pro-mitotic signals include the Insulin/IGF signaling pathway (IIS), which is sufficient and required for ISC proliferation [18], [23], [33], [34]. Activation of the Insulin Receptor (InR) in flies initiates an evolutionarily conserved signaling cascade composed of insulin receptor substrate (IRS, Chico), PI3Kinase (DP110) and Akt, inducing cell proliferation and/or growth and endoreplication [35]–[39]. Interestingly, IIS induces ISC proliferation through both cell-autonomous mechanisms involving the Akt-regulated transcription factor Foxo, as well as through a non-autonomous process in which IIS – induced EB differentiation is critical to allow further ISC divisions [7], [34]. In EBs, InR is sufficient and required for differentiation into ECs [34].

In most *Drosophila* tissues, cell growth is regulated downstream of Akt by the evolutionarily conserved TSC/Rheb/TOR pathway [37], [40], [41]. As supported by genetic and biochemical studies, this pathway can be activated in response to Akt-mediated phosphorylation of Tuberous Sclerosis Complex 2 (TSC2; encoded by the gene *gigas* in *Drosophila*) and subsequent inhibition of the TSC1/2 complex [37], [40]. TSC1 promotes the stability of TSC2, which is a GTPase activating protein for the small GTPase Rheb, inhibiting Rheb-mediated TOR Kinase activation. TOR, in turn, phosphorylates translational regulators, including ribosomal protein S6 Kinase (S6K) and eIF4E Binding Protein (4EBP), resulting in a net increase of protein production in cells.

In addition to Akt-mediated phosphorylation, other signals are likely to play an important role in regulating TSC1/2 activity *in vivo* [42], [43]. Accordingly, various other regulatory events, including phosphorylation in response to several growth factors and morphogens, ubiquitination, and degradation, have been reported to influence TSC1/2 activity [37], [40], [44]. The TSC1/2 complex thus represents a critical node in signaling networks that arbitrate between cell proliferation and growth in response to increased insulin signaling. Supporting this view, mutations in TSC1/2 result in Tuberous Sclerosis Complex, a rare autosomal dominant disease that is characterized by widespread benign tumor formation [45].

Recent studies suggest that TSC/TOR signaling has an important regulatory role in both vertebrate and invertebrate stem cell lineages. In human embryonic stem cells, activation of S6K by mTOR has been reported to induce differentiation [46], while a recent study in the mouse has identified an interesting non-autonomous function for mTOR activity in ISC support cells, the Paneth cells. Under conditions of dietary restriction, TOR signaling activity is reduced in Paneth cells, resulting in secretion of factors that promote stem cell maintenance and proliferation [47]. In the *Drosophila* germline, TSC/TOR signaling regulates proliferation and maintenance of germline stem cells (GSCs) [48]–[50]. GSCs mutant for TSC1/2 undergo differentiation, through a so far unknown mechanism [49], [50], while GSCs mutant for the TOR kinase exhibit proliferation defects [49]. TSC/TOR signaling is thus likely to mediate, at least partially, the effects of the dietary status of the organism on GSC proliferation and maintenance [48]–[50] [4], [51].

In the *Drosophila* intestine, TSC/TOR signaling may have a similar function, as ISCs are also regulated according to nutrient availability [33], [52]. Indeed, a recent report shows that loss of TSC in ISCs causes excessive ISC growth and impairs ISC proliferation [53]. Using the ISC and EB driver esgGal4, it was shown that TSC2-RNAi expressing ISCs become large, express less cell cycle markers, have reduced DNA replication, and that these phenotypes are Rapamycin-sensitive. These cells further fail to respond to tissue damage by initiating cell divisions, and exhibit increased DNA content, indicating that they are becoming polyploidy [53]. While these characteristics indicated differentiation of TSC deficient cells, it was shown that TSC2-RNAi expressing cells do not express the EC marker Pdm1, and do not form ECs with brushed borders, suggesting that they may have initiated, but not completed, differentiation. TSC-mediated inhibition of TOR signaling thus seems to be critical to maintain ISC activity and function. It remained unclear, however, what physiological role, if any, TOR activation may have in ISCs or their daughter cells, and how Tor signaling may interact with others pathways regulating ISC commitment and differentiation.

Here, to address these questions, we characterize the function and regulation of TSC/TOR signaling in the ISC lineage in more detail. We find that TSC2 is highly expressed in ISCs, but specifically down regulated in EBs. While, consistent with the previous report [53], high TSC2 expression is required for ISC function, we also find that the down-regulation of TSC2 in EBs, and the resulting TOR activation, are critical for EC differentiation. Our results further suggest that TSC activity promotes lineage commitment of EBs into the EE fate.

To characterize the regulation of TSC/TOR signaling in EBs further, we assessed its interaction with the Notch (N) signaling pathway. We find that N-induced Su(H) activity represses TSC2 expression in EBs. Strikingly, repression of TSC1/2 function is sufficient to commit cells into the EC fate independently of N activity, indicating that TSC2 repression is a central step in N-induced EC differentiation.

We also find that food conditions significantly impact the proliferative capacity of TSC-deficient ISCs. We show that TSC mutant ISCs are capable of generating normal clones of daughter cells on a low calorie (low yeast) diet, but that these lineages decline over time. Rearing flies on high-yeast food, however, causes growth and proliferation phenotypes similar to the ones observed in [53], accelerating the decline of TSC mutant clones. TSC activity in ISCs is thus specifically required to maintain ISC function under high nutrient conditions.

## Results

### Regulation of cell proliferation and growth by TOR signaling in the *Drosophila* intestine

While InR/Akt signaling can activate TOR signaling in many *Drosophila* tissues [35]–[37], [40], [41], previous reports have suggested an opposing role of InR and TOR signaling in the control of ISC proliferation: While Insulin/IGF signaling (IIS) is required for ISC proliferation, and activation of IIS (by InR over-expression) induces increased proliferation [18], [23], [33], [34], activation of TOR signaling (by loss of TSC function) was found to impair proliferative capacity [53]. Using RNAi-based knockdown of TSC2 in ISCs and EBs, Amcheslavsky et al found that loss of TSC2 increased the size of ISCs. Based on cell cycle markers and EdU incorporation experiments, it was concluded that these cells are not mitotically active. Furthermore, the proliferative capacity of TSC2 homozygous mutant ISCs was assessed using lineage tracing by somatic recombination. However, a mitotic recombination with a repressible cell marker (MARCM, [54]) approach was used in which GFP was expressed under the control of esg::Gal4, which labels only ISCs and EBs (see Materials and Methods in [53]). Clones with more than two cells (including ECs and EEs) that may be formed by TSC mutant ISCs (see below) can not be observed with this approach (see for example Fig. 2A in [53]), and a full lineage analysis of TSC deficient ISCs was thus not possible. While the results reported in [53] thus clearly identified a critical role for TSC2 in maintaining small, diploid ISCs, it remained unclear whether activation of TOR signaling in ISCs would fully impair their proliferative activity and prevent generation of ISC daughter cells. Interestingly, the reported results indicated that TSC2 function is required in ISCs for IIS-mediated induction of proliferation, suggesting that IIS activation does not result in inactivation of TSC in the ISC lineage. It further remained unclear whether TOR signaling has to be continuously repressed by TSC2 in the ISC lineage, or whether TOR activation occurs naturally in the lineage to regulate proliferation, growth or differentiation of ISCs or their daughter cells.

**Figure 1:**
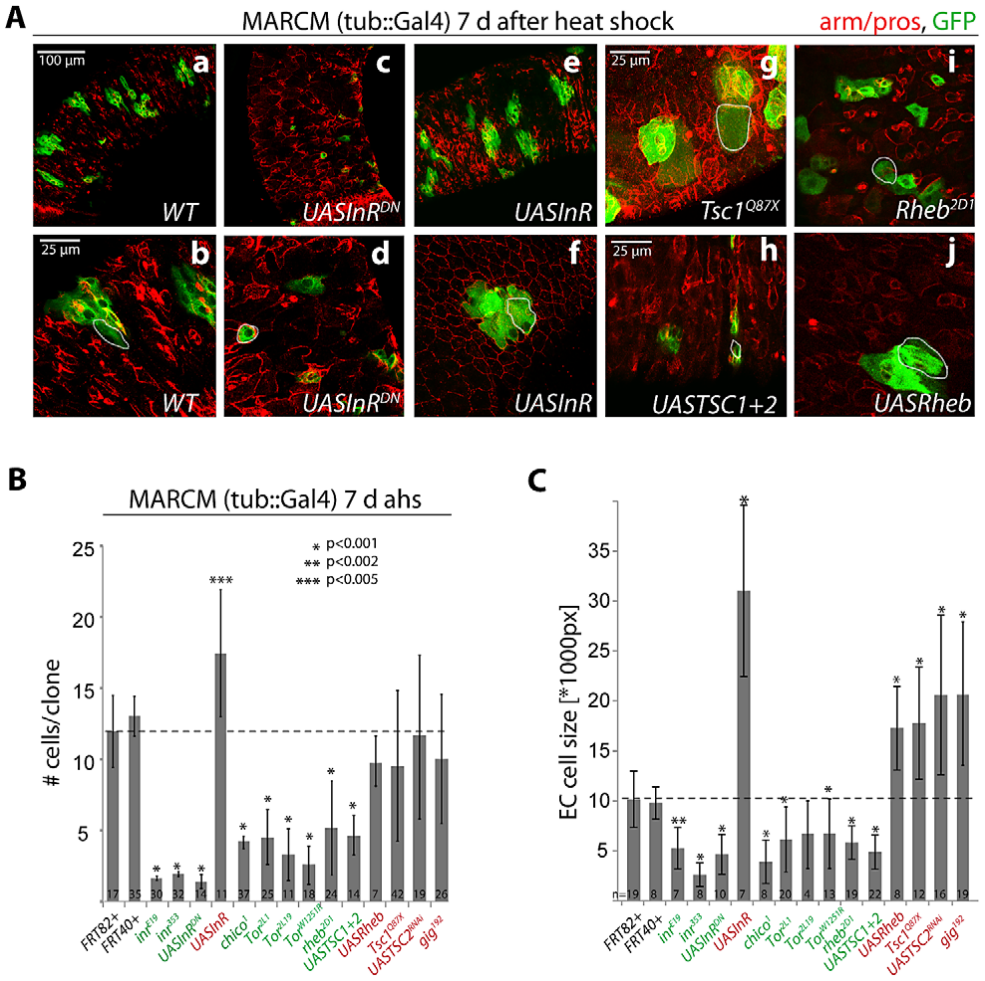
Regulation of proliferation and growth in the ISC lineage by the InR/TOR pathway. A: Confocal images of posterior midgut epithelia containing GFP-labeled MARCM clones of the indicated genotypes at 7 days after heat shock (d ahs). Armadillo and prospero antibodies (red) detect cell outlines and EE cells, respectively. GFP, green. B, C. Quantification of MARCM clone size (# cells/clone) at 1 and 7 d ahs, and EC cell size at 7 d ahs (Dl antibody staining was used to distinguish ISCs from other cells. Size of the largest Dl− cells in each clone was measured, compare with white outlines in A). Loss of Rheb did not reduce clone size at 1 d ahs, but it is unclear if at this time Rheb protein levels have already been decreased in these cells. Averages and standard deviations are shown; Student's T-test was used to assess statistical significance. Numbers of analyzed clones (N) are shown in the bars.

To characterize the relationship between InR and the TOR signaling pathway in ISC lineages in more detail, we generated ISC clones with gain- and loss-of-function conditions for multiple IIS and TOR pathway components (Figure 1). We used MARCM to generate ISC clones that over-express wild-type or dominant-negative insulin receptor (InR; [55]) molecules, that were mutant for the IRS homologue Chico (carrying the loss of function allele *chico^1^* [56]), or that were homozygous for the *InR* loss-of-function alleles *InR^E19^* or *InR^353^* [57]. Similarly, we generated clones with TOR pathway gain- and loss-of-function conditions by over-expressing Rheb or TSC1 and 2, introducing the *TSC1* loss of function allele *Tsc1^Q87X^* [58], the *TSC2* loss of function allele *gigas^192^* [59], the TOR loss of function alleles *Tor^2L1^*, *Tor^2L19^* and *Tor^W1251R^* [60], or the *Rheb* loss of function allele *Rheb^2D1^* [61], or expressing dsRNA against TSC2 (TSC2^RNAi^).

Importantly, we used a MARCM approach in which all daughter cells of mutant ISCs are labeled by GFP (since GFP was expressed under the control of tub::Gal4). The vast majority of GFP+ lineages (>95%) in the midgut were induced by mitotic recombination in response to heat shock, as very few marked cell clones could be observed in control animals (Figure S1C). The number of cells in each clone at a given time point after the heat shock thus accurately reflects ISC proliferation.

Consistent with previous reports [18], [23], [33], [34], gain of InR function increased the number of cells produced in a clone, while loss of *InR* or *chico* activity significantly reduced the number of cells produced by an ISC in 7 days (Figure 1A, 1B). Loss of TOR pathway activity also reduced clone sizes at 7 days, and increasing TOR pathway activity (in *TSC* mutants or Rheb over-expressing clones) resulted in clones that showed no significant difference in average cell numbers at 7 day after induction. In contrast to the observations reported in [53], ISCs with increased TOR pathway (i.e. reduced TSC) activity were thus capable of generating normal ISC lineages in our studies. However, the variability in clone sizes increased in TOR gain of function conditions compared to wild-type clones (compare standard deviations in Figure 1B), indicating that, consistent with [53], individual ISCs may lose the ability to generate normal numbers of daughter cells (see below). As expected, we also observed significantly larger Enterocytes (ECs, defined as the largest polyploid, Dl - negative cell in a clone) in both IIS and TOR gain-of-function conditions at 7 days after clone induction, and significantly smaller cells in IIS/TOR loss-of-function conditions (Figure 1C). This is consistent with previous findings in developmental contexts and in GSCs, showing that IIS and TOR signaling act in concert to promote endoreplication and growth [39], [49].

Our results thus support a positive interaction between InR and TOR signaling in the ISC lineage. We tested whether TOR signaling acts downstream of InR in the regulation of proliferation and growth in this lineage by assessing the frequency of mitotic figures and the size of EC nuclei. We co-overexpressed InR with TSC1 and TSC2, or with dsRNA against S6K (S6K^RNAi^) using the ISC/EB driver esg::Gal4 in combination with the heat-sensitive Gal4 inhibitor Gal80^ts^ (Figure S1A, S1B; TARGET system [8], [9], [62]). InR over-expression using this driver dramatically increases ISC proliferation rates (as represented by the number of phospho-histone H3 (pH3) positive cells [18]) and increases cell sizes in the gut (as represented by the size of EC nuclei; Figure S1A, S1B). Loss of TOR pathway activity (over-expression of TSC1 and 2 or knockdown of S6K) did not affect InR-mediated proliferation, but significantly prevented the increase in EC nuclear size. In these InR gain-of-function conditions, the TSC/TOR/S6K pathway is thus specifically required to promote growth and endoreplication rather than proliferation in the ISC lineage.

### Nutrient-dependent phenotypes of TSC deficient ISCs

**Figure 2:**
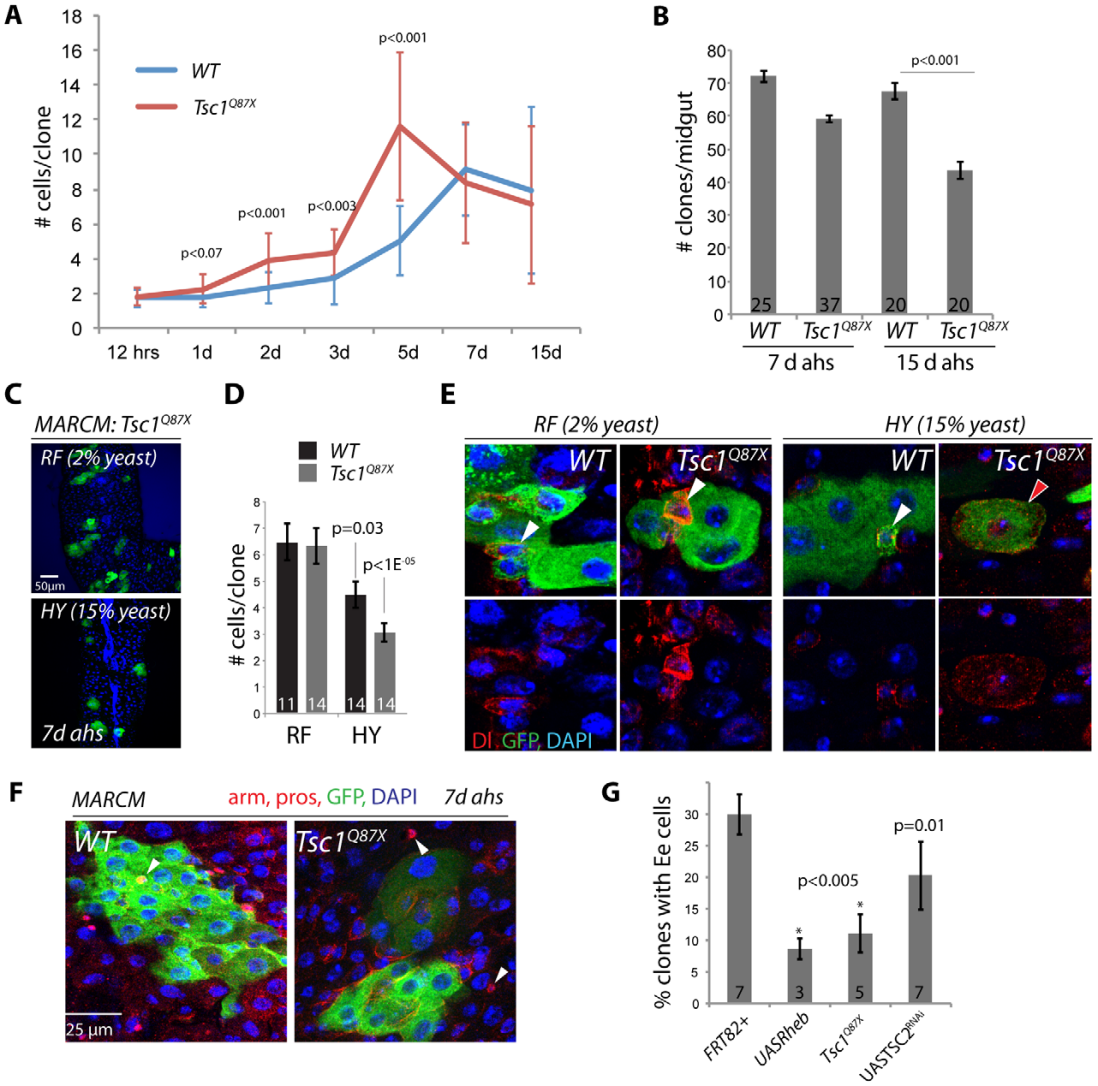
Nutrient-dependent dysfunction of TSC mutant ISCs. A. Timecourse analysis of MARCM clone growth. *Tsc1^q87x^* mutant and wild type clones were induced at 3 days of age by a 45 min heat shock. The number of GFP labeled cells in each clone was determined for 5–10 guts and 30–50 clones for each timepoint. Averages and STDEV are shown. P values from Student's T-test. B. TSC1 mutant clones are progressively lost from the intestinal epithelium. Number of MARCM clones observed in each midgut at 7 and 15 d ahs is shown (Averages and SEM; Student's T-test). C, D. TSC1 mutant ISCs become dysfunctional in high-calorie food. TSC1 (*Tsc1^Q87X^*) mutant ISCs from flies fed regular food (RF, 2% yeast) form larger clones (more cells per clone) compared to TSC1 mutant clones from flies fed high calorie food (HY, 15% yeast). High-yeast food also reduces clone sizes of wild-type ISCs somewhat, but these are not significantly different from wild type (compare p values determined between high-yeast and regular food conditions for each genotype). Averages and SEM are shown in this quantification. E. In most TSC mutant clones maintained on HY, Dl+ ISCs become large (right panels), while mutant clones maintained on RF retain small diploid ISCs. In wild-type clones, Dl+ cells are small and diploid independently of the food conditions. Dl red, GFP, green, DAPI, blue. F, G. MARCM clones deficient for TSC1 (*Tsc1^Q87X^*) or TSC2 (*TSC2^RNAi^*), or over-expressing Rheb (UAS-Rheb) contain EE cells significantly more rarely than wild-type clones. Example clones are shown in F. EE cells identified by anti-pros staining (arrowheads; Armadillo and prospero red, GFP green, and DAPI blue). Quantification in G (Averages and SEM. Student's T-test. The number of guts in which the percentage of EE containing clones was quantified is shown in each bar.

Since these results contrasted with the observations reported in [53], we assessed the phenotypes of TSC deficient ISCs in more detail. A timecourse analysis revealed that loss of TSC1 resulted in clones that initially grew faster than wild-type clones, but declined and became heterogeneous in size at later timepoints (Figure 2A and Figure S2A; see large standard deviations in TSC1 mutant clones at 5, 7, and 15 days, and compare with TSC2 mutant clones in Figure 1B). This indicated an initial increase of proliferative activity in TSC mutant ISCs, followed by a sporadic loss of proliferation in individual ISCs at a later timepoint. Indeed, while many *Tsc1^Q87X^* or *gigas^19^* ^2^ mutant clones, or clones expressing TSC2^RNAi^, were recovered that contained a single diploid Dl+ ISC even at 15 days after clone induction, at all ages rare clones could also be observed in which the Dl+ cell became large and polyploid, consistent with the phenotype reported by Amcheslavsky et al (Figure S2B, S2C). In our experiments, TSC mutant ISCs did thus not immediately increase in size, but grew and lost function sporadically. This interpretation is supported by the fact that the number of *Tsc1^Q87X^* mutant clones observed in the gut declined over time (Figure 2B).

To explore why TSC mutant ISCs exhibited a much less penetrant growth phenotype in our experiments as compared to [53], we tested whether the rate of the spontaneous growth of TSC mutant ISCs might be influenced by dietary conditions, which can modulate TOR activity independently of TSC [35]–[39]. Indeed, the average number of cells generated by *Tsc1^Q87X^* mutant ISCs within 7 days was significantly reduced when flies were reared on high yeast food (HY, 15% yeast) compared to our regular food (RF, 2% yeast)(Figure 2C, 2D). Dl+ cells in these *Tsc1^Q87X^* mutant clones became large and polyploid, similar to the phenotype described in [53](Figure 2E). Clone sizes were also reduced in wild-type flies reared on high yeast food, but this reduction was less significant than the size reduction of TSC deficient clones (Figure 2D).

Two recent studies have reported strong effects of yeast, the only protein source in fly food, on ISC activity. Both studies reported increased ISC activity in yeast-fed flies compared to flies completely starved of yeast [33], [34]. ISCs thus require a protein source to become fully active, yet our results indicate that they can also lose function when protein levels are too high. This effect is significantly enhanced when TSC is lost, indicating that TSC activity isolates the TOR pathway from dietary stimuli in ISCs, maintaining their function. The role of the TSC1/2 complex in ensuring the long-term maintenance of ISCs is thus reminiscent of its function in GSCs [48]–[50].

Interestingly, *Tsc1^Q87X^* mutant, or TSC2^RNAi^ or Rheb over-expressing clones were significantly less likely to contain prospero-labeled EE cells than wild-type clones, suggesting that TOR activation also impaired the commitment of EBs into the EE cell fate, or the terminal differentiation of EEs (Figure 2F, 2G). It remains unclear, however, whether this is a consequence of direct TOR pathway-mediated regulation of prospero expression, or of other events required for EE differentiation.

Our observations thus suggest that the TSC complex promotes ISC maintenance in varying nutritional conditions, influences commitment into the EE fate, and regulates EC growth in the intestinal epithelium.

### Notch-mediated suppression of TSC2 expression in ISCs/EBs

**Figure 3:**
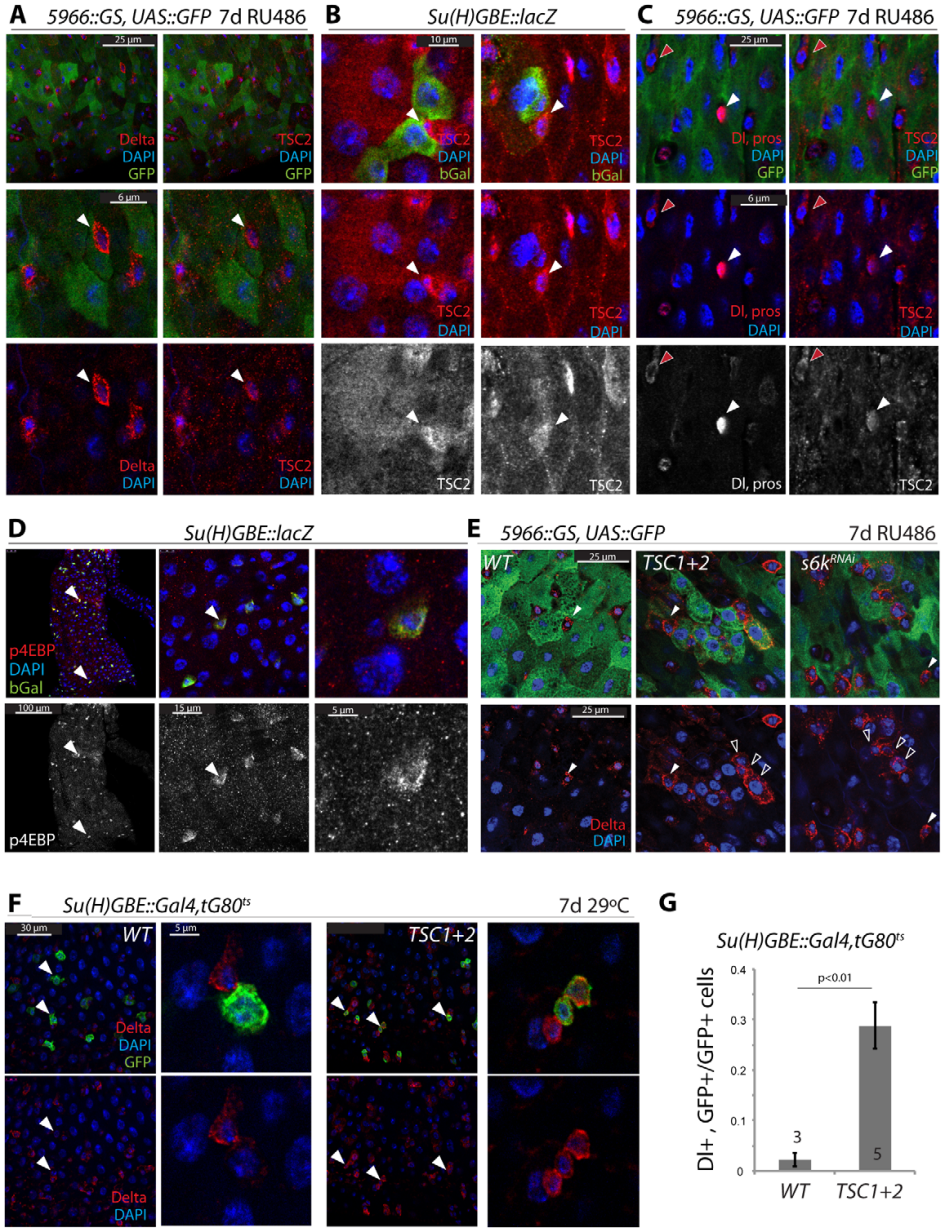
Transient repression of TSC2 expression in the ISC lineage. A. TSC2 is highly expressed in GFP−, Dl+ ISCs (arrowheads) of 5966GS>GFP flies. Confocal images detecting TSC2 (red, Tritc channel, right panels) and Dl (red, Cy5 channel, left panels) were acquired using sequential scanning to separate Cy5 and TRITC channels. False-color images were composed from individual channels in Photoshop. 5966GS drives UAS::GFP (green) in EBs and ECs, but not in ISCs. Flies were exposed to RU486 for 7 days. B. TSC2 expression is reduced in EBs. Su(H)-GBE::lacZ drives bGalactosidase expression specifically in EBs. b-gal, green; TSC2, red; DAPI, blue. Arrowheads point to individual ISCs. TSC2 channel is shown separately in bottom panels. C. TSC2 expression in EEs and ISCs. TSC2, red, right panels; Dl and pros, red, left panels; DAPI, blue. Dl, pros, and TSC2 channels are shown separately in bottom panels. Red arrowheads point to a selected ISC; white arrowhead to a selected EE. D. High levels of phosphorylated 4EBP are detected in Su(H)+EBs. Anti-p4EBP, red/white; anti-bGalactosidase, green. E. Over-expression of TSC1 and 2, or S6K^RNAi^ in EBs and ECs impairs EB differentiation. E: Dl staining in flies expressing GFP, TSC1+2, or S6K^RNAi^ under the control of *5966::GS*. Flies were exposed to RU486 for 7 days. Closed arrowheads point to GFP−, Dl+ ISCs, open arrowheads to GFP+, Dl+ EBs. F, G. Over-expression of TSC1 and 2 in EBs impairs EB differentiation. F: Dl staining in flies expressing GFP and TSC1+2 under the control of *Su(H)-GBE::Gal4; tub::Gal80^ts^*. Flies were reared at 29°C for 7 days. Arrowheads point to selected GFP+/Dl+ cells. G: quantification (Averages and SEM) of the fraction of GFP+ cells that were also Dl+. The number of guts quantified is shown in the bars. The total number of cells counted was 240 (wt) and 340 (TSC1/2 overexpression).

We hypothesized that these multiple functions of TSC are coordinated by intricate, cell-type specific regulation of TSC activity in the ISC lineage. To start analyzing this regulation, we examined the expression of TSC2 using an anti-Gigas antibody described in [44]. High expression of TSC2 was detected in ISCs (Dl+ cells that do not express GFP under the control of the RU486-inducible EB/EC driver *5966::GS* [63], Figure 3A) and in EEs (pros+ cells, Figure 3C. These cells show even higher TSC2 expression than ISCs), and its expression was significantly weaker in EBs (cells expressing bGalactosidase from a Su(H)-GBE::lacZ construct, Figure 3B). Consistent with this expression pattern of TSC2, we found that in wild-type homeostatic conditions, the TOR pathway is highly active in EBs (compared to ISCs or ECs), as determined using an antibody against phosphorylated 4EBP (Figure 3D, this antibody reliably detects changes in TOR signaling activity, see S3A and [49]). Preventing this activation of TOR signaling in EBs was sufficient to impair the formation of normal EBs: over-expression of TSC1/2 or knockdown of S6K (S6K^RNAi^) specifically in EBs and ECs (using *5966::GS*), resulted in the accumulation of small Dl+ cells that also express GFP (Figure 3E). Most of these cells had DNA content that was similar to ISCs, indicating that they are diploid or have not completed endoreplication (Figure S3B). The disruption of the normal asymmetric distribution of Dl in ISC/EB pairs indicates that TOR inactivation in ISC daughter cells inhibits differentiation. A similar disruption of normal EB determination was observed when TSC1/2 were over-expressed in EBs only using *Su(H)-GBE::Gal4* [64] (Figure 3F, 3G). Interestingly, these guts also exhibited a significant increase in the number of pros+ EE cells, indicating that inhibiting TOR activity in EBs is sufficient to alter their commitment from the EC fate into the EE fate (Figure S3C).

**Figure 4:**
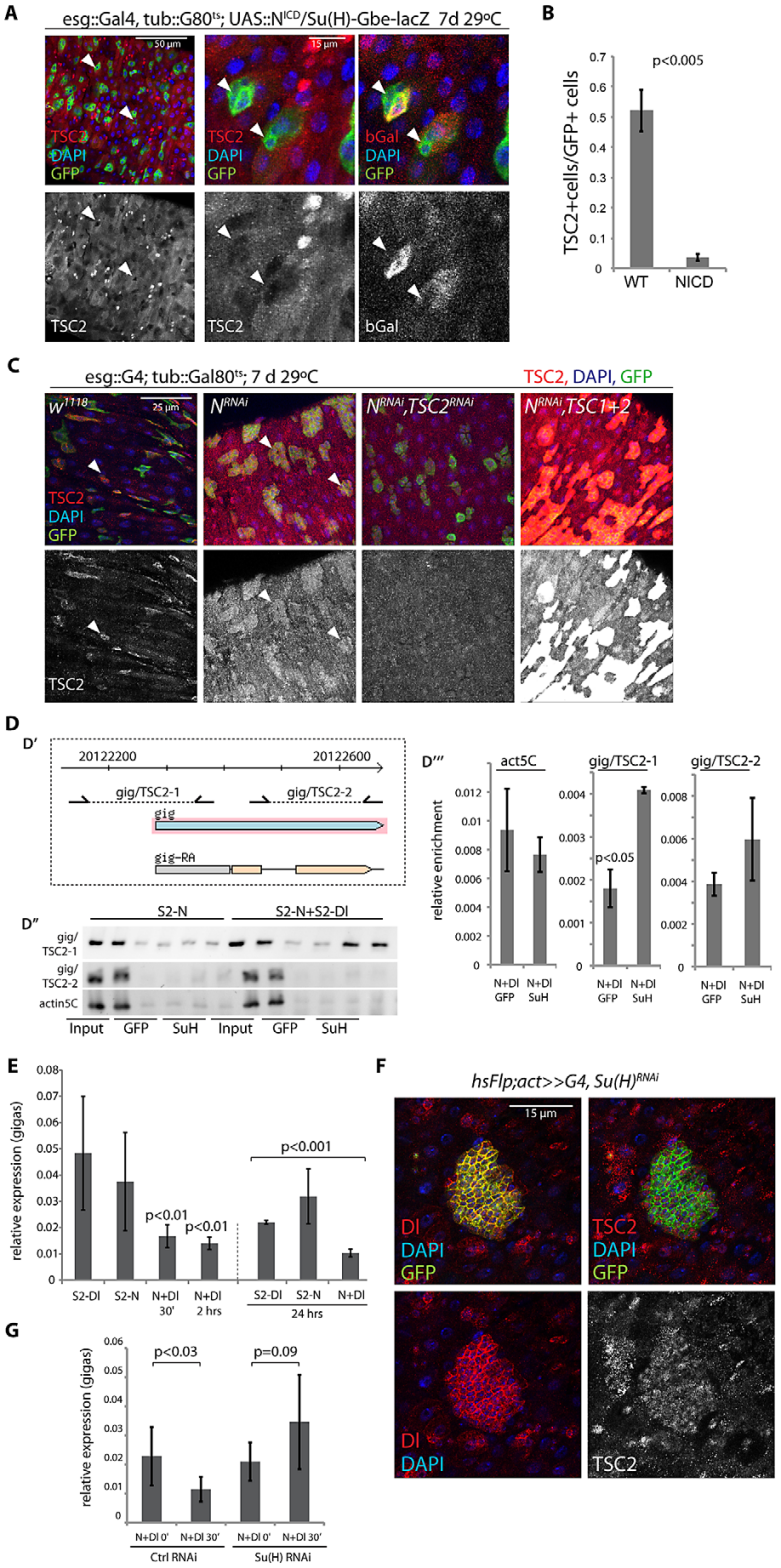
Suppression of TSC2 expression in response to Notch activation in EBs. A, B. Over-expression of N intracellular domain (N^ICD^; using esg::Gal4, tub:Gal80^ts^ combined with the N reporter Su(H)-Gbe::lacZ) decreases TSC2 levels in stem cells (arrowheads). A: Confocal micrographs. GFP (ISC and EB), green; TSC2, red (left and middle); b-gal (EB), red (right panel); DAPI, blue. B: Quantification of the fraction of esgGFP+ cells that are also TSC2+ (Averages and SEM, Student's Ttest. N = 3 guts each, between 20 and 115 GFP+ cells were quantified in each gut). C. Expressing N^RNAi^ under the control of esg>Gal80^ts^ causes ISC tumors (left center panels). Cells in these tumors retain high TSC2 levels. Knocking down TSC2 by TSC2^RNAi^ causes most GFP marked cells to loose TSC2 staining (right center panel), while TSC1 and 2 over-expression in this background results in a strong positive signal (right panels), confirming the specificity of the antibody. D. Chromatin IP to detect Su(H) binding to the gigas promoter. D′. Diagram showing location of primer sets (gig/TSC2-1 and gig/TSC2-2) relative to the *gigas/Tsc2* transcriptional start site. Coordinates are from *Drosophila* genome release 5. D″. PCR on immunoprecipitates from S2 cells containing Cu^2+^ inducible Notch expression constructs or from co-cultures of N-expressing and Dl-expressing S2 cells. Precipitates with GFP antibodies (negative control) and with Su(H) antibodies are shown relative to input (non-precipitated, sheared DNA). D′″. Real-time PCR to quantify enrichment relative to input in samples of N/Dl co-cultures. Averages and standard deviations (N = 3), Student's Ttest. E. Real-time RT-PCR assessing *gigas/Tsc2* mRNA levels in S2 cell co-cultures. *Gigas/Tsc2* transcript levels relative to *actin5C* are shown at indicated timepoints after initiating co-culture. Averages and standard deviations (N = 3), Student's Ttest. F. Flp-out clones expressing Su(H)^RNAi^ consist of small Dl+ cells that retain high TSC2 expression. RFP (flp-out clone), green; Dl, red (left panels); TSC2, red (right panels); DAPI, blue. G. Treating N-expressing S2 cells with Su(H) RNAi before initiating co-culture prevents repression of *gigas/Tsc2*. Averages and standard deviations, N = 6; Student's Ttest.

Combined, our findings suggested that reduced TSC1/2 function in EBs is critical for differentiation of EBs and for lineage commitment into the EC fate. Importantly, these findings also suggested a potential mechanism for TSC2 regulation in the ISC lineage, as down-regulation of TSC2 expression coincides with the activation of N signaling in EBs. We hypothesized that N activation promotes TSC2 down-regulation and tested this idea by over-expressing the N Intracellular Domain (N^ICD^) in ISCs and EBs (using esg::Gal4, Gal80^ts^). Expression of N^ICD^ is sufficient to force differentiation of ISCs into ECs [8], [9]. Consistently, we found that TSC2 expression was undetectable in most esg::GFP+ cells expressing NICD, while in wild-type intestines, more than 50% of all esg::GFP+ cells express high levels of TSC2 (Figure 4A, 4B). We further tested whether N signaling is required for TSC2 repression in the ISC lineage by over-expressing a dsRNA against N (N^RNAi^) in ISCs and EBs. Expression of N^RNAi^ under the control of esg::Gal4 prevents EB differentiation and results in the formation of ISC tumors characterized by clusters of small, diploid, Dl^+^ cells [8], [9]. Cells in these tumors were also TSC2 positive, confirming the correlation between ISC identity and TSC2 expression, and suggesting that N signaling is required for TSC2 repression (Figure 4C; TSC2 immunoreactivity was suppressed by TSC2^RNAi^ and enhanced by over-expressing both TSC1 and 2, confirming the specificity of the antibody. Co-expression of TSC1/2 also moderately increased the size of the stem cell tumors, indicating additional enhancement of the N^RNAi^-caused phenotype).

The N-responsive transcriptional regulator Su(H) has been reported to bind to a cluster of four sites within 1.5 kb in the upstream promoter region of the *gigas*/*Tsc2* gene in *Drosophila* [65]. Su(H)-mediated transcriptional repression of *gigas*/*Tsc2* was thus a plausible mechanism for N-induced repression of TSC2 expression in EBs. To test this idea, we assessed the regulation of *gigas/Tsc2* in co-cultures of S2 cells that constitutively express N or Dl [66](Figure 4D–4E; N activation in N-expressing cells occurs within minutes of exposure to Dl-expressing cells, Figure S4). We first confirmed that Su(H) binds to the upstream promoter region of gigas/Tsc2 using chromatin IP (ChIP, Figure 4D), and found significant enrichment of a region proximal to the transcriptional start site in precipitates from cells with activated N signaling. We further measured transcript levels of *gigas/Tsc2* and found reduced expression of this gene within 30 min of N activation (Figure 4E). This repression of *gigas/Tsc2* was sustained for at least 24 hours. Protein levels of TSC2 (measured by Western Blot) did not decrease significantly in S2 cells in these experiments (not shown), indicating that in addition to transcriptional repression, posttranslational mechanisms have to be involved in reducing TSC2 protein levels *in vivo* as observed in ISCs expressing N^ICD^ (Figure 4A, 4B). Importantly, these results suggested that Su(H) is a general transcriptional repressor of *gigas/Tsc2* expression in *Drosophila* cells. Accordingly, TSC2 repression in EBs was mediated by Su(H), as inducing ‘Flp-out’ clones [67] expressing Su(H)^RNAi^ was sufficient for the formation of tumors containing small Dl+ and TSC2+ cells (Figure 4F). Consistently, *gigas/Tsc2* repression in the S2 co-culture system was prevented when Su(H) was knocked down by RNAi (Figure 4G).

### TSC/TOR signaling regulates EC differentiation

**Figure 5:**
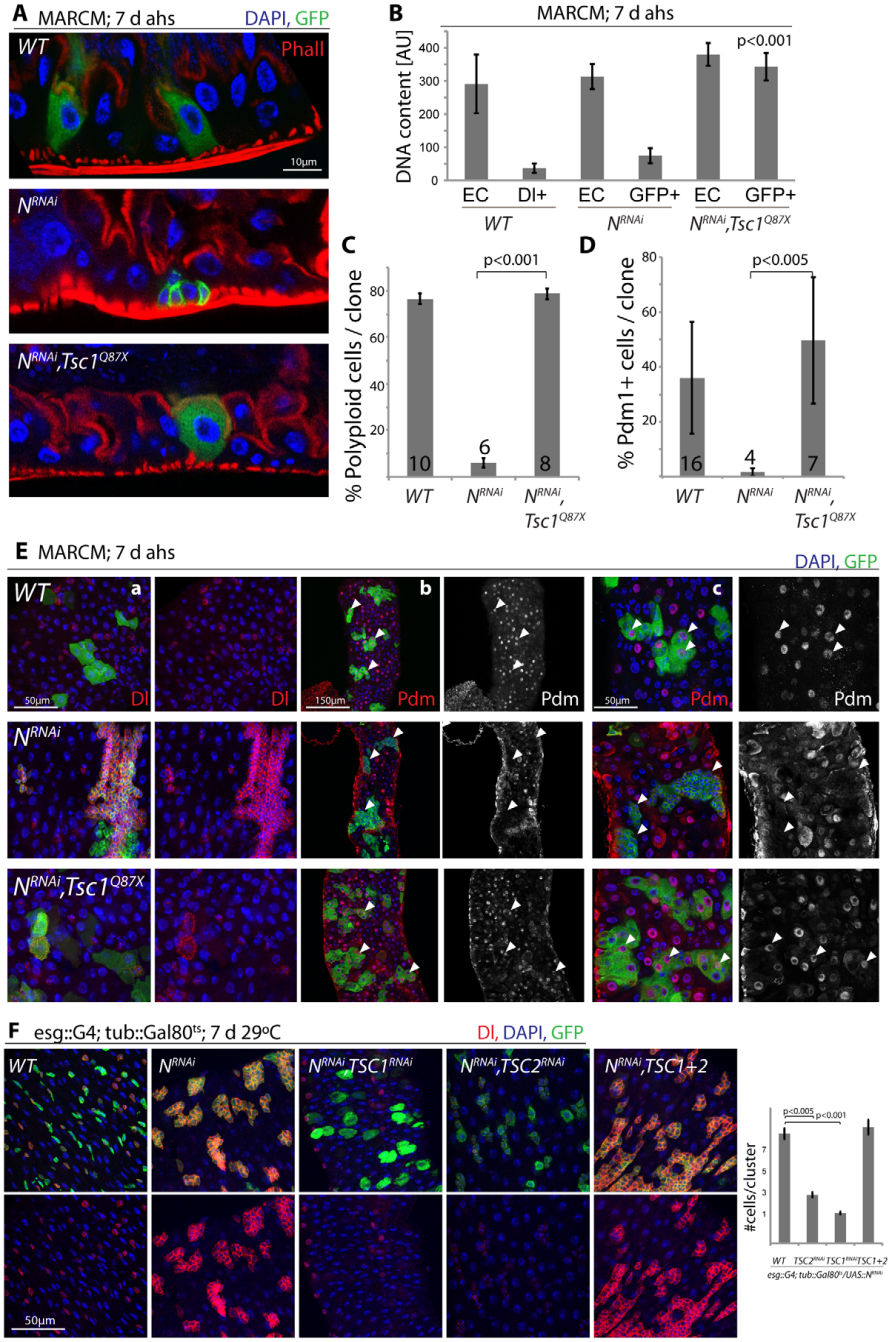
TSC repression in EBs is sufficient for ISC differentiation. A. Loss of TSC1 (*Tsc1^Q87X^*) rescues ISC tumor formation in a N^RNAi^ expressing background. MARCM clones; cell sizes in N, TSC1 double-mutant lineages are almost identical to wild-type clones, and ECs differentiate that acquire a brush border. GFP (MARCM clones), green; Phall (Phalloidin, highlighting the brush border), red; DAPI, blue. B. Loss of TSC1 induces polyploidy in N^RNAi^ loss-of-function background. Cells in MARCM clones deficient for TSC1 (*Tsc1^Q87X^*) and N^RNAi^ have DNA content similar to wild-type ECs, compared to N^RNAI^ alone in which DNA content remains similar to wild-type ISCs. DNA content was analyzed by integrating DAPI intensity values across all pixels for individual nuclei using TCSNT software from Leica (arbitrary units [AU] are listed). ECs (outside of clone) and small Dl+/GFP+ cells (ISCs, within clones) were measured for WT, while EC (outside of clone) and GFP+ cells within clones were measured for N^RNAi^ and N^RNAi^, *Tsc1^q87x^*. Bar represent averages and standard deviations (N = 5–10 nuclei, from 3 independent guts), Student's Ttest. Nuclear sizes of WT/Dl+ cells and of N^RNAi^/GFP+ cells show little variability, allowing precise quantification using small numbers of representative nuclei. See also quantification in C and images in E. C. The fraction of polyploid cells (defined as any cell with a nucleus that is larger than the smallest Dl+ cell) in ISC clones decreases dramatically in N loss of function conditions, and is rescued when *Tsc1* is also lost. Bar represent averages and standard deviations (N shown in each bar), Student's Ttest. D, E. Expression of Dl is reduced, while expression of *pdm1* is restored to wild-type levels in N, *Tsc1^q87x^* double-mutant lineages, confirming the loss of ISC identity and the acquisition of EC identity. Quantification of the fraction of Pdm1+ cells in clones is shown in D (Averages and standard deviations, N shown in each bar; Student's Ttest), confocal micrographs of Dl (red, panels a) and Pdm1 antibody staining (red and white, panels b and c) in E. GFP green, DAPI blue. White arrowheads point to individual clones, highlighting the absence of Pdm1+ cells in N loss of function conditions and the restoration of Pdm1+ ECs when *Tsc1* is lost. While most N^RNAi^/*Tsc1^Q87X^* cells become polyploid, have a brush border and are Dl−, occasionally large Dl+ cells can be observed (as seen in one clone in a). F. Intestines expressing N^RNAi^ and TSC1 or 2^RNAi^ under the control of esg>Gal80^ts^ for 7 days. Note that loss of TSC1 or 2 reduces Dl expression in N^RNAi^-expressing ISCs and EBs, indicating differentiation of ISCs. GFP (ISCs and EBs), green; Dl, red; DAPI, blue. Quantification of the sizes of individual stem cell clusters is shown on the right. Averages and SEM; Student's Ttest. N = 7 guts each, 15–40 clusters/gut.

These findings are consistent with a model in which N activation suppresses TSC2 expression in EBs, inducing growth and endoreplication in response to insulin signals. We asked whether TSC2 repression was sufficient and required for ISC differentiation downstream of N, and found that loss of TSC1/2 indeed rescued the tumor phenotype of N^RNAi^ expressing ISCs (in both MARCM clones, and when driven by esg::Gal4; Figure 5, Figure S5). N-deficient ISCs generate tumors because they undergo symmetric divisions and thus generate exponentially growing cell clones. Loss of TSC1/2 prevented this accumulation of Dl+ ISCs in N loss of function conditions and converted N^RNAi^ expressing cells into Dl−, polyploid, EC-like cells. Similar to wild-type ECs, these cells also contained brush borders and expressed the EC marker Pdm1 (Figure 5A–5E, Figure S5A–S5C; brush borders can be observed by staining for phalloidin; Polyploidy measured by intensity of DAPI fluorescence). TSC1 suppression is thus sufficient to fully differentiate N-deficient ISCs into ECs. Consistent with a conversion of these cells into a postmitotic state, the number of cells observed in each cluster of N^RNAi^ expressing cells was significantly reduced when TSC1 or 2 were lost (Figure 5E, 5F).

These results confirm that loss of TSC1/2 in N loss-of-function conditions is sufficient to promote differentiation of ISCs towards the EC fate. For most analyzed phenotypes, inhibition of TSC1 elicited stronger effects than inhibition of TSC2, suggesting that the knockdown of TSC2 is less efficient, or reflecting the fact that loss of TSC1 also results in degradation of TSC2 protein, as TSC1 stabilizes TSC2. While many TSC1/Notch double mutant cells thus are morphologically indistinguishable from wild-type ECs, it is important to note, however, that Notch activation elicits complex gene expression changes in cells, and it remains unclear whether all functional aspects of ECs can be reconstituted in N/TSC1/2 deficient cells. Furthermore, some of these cells retain Dl expression (see example in Figure 5E), indicating that not all of these cells fully differentiate into normal ECs.

**Figure 6:**
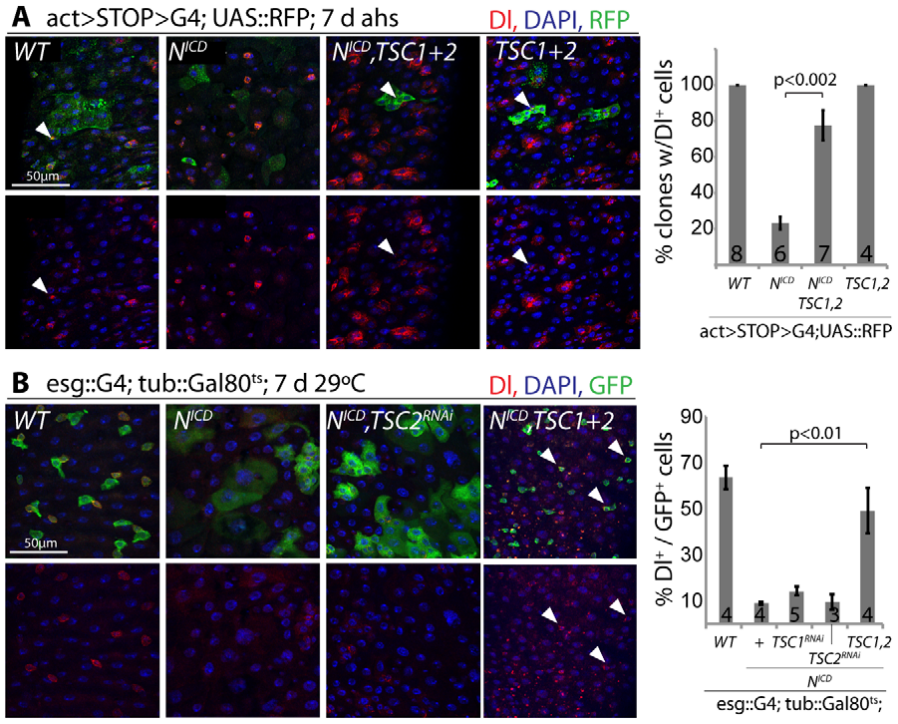
TSC repression in EBs is required for ISC differentiation. A. Flp-out-mediated over-expression of N^ICD^ promotes ISC differentiation (small clones with large, Dl− cells are formed). Co-expression of TSC1 and 2 rescues this phenotype. RFP (clone), green; Dl, red; DAPI, blue. The percentage of clones containing at least 1 Dl+ cell is quantified on the right (Averages and SEM; Student's Ttest; N shown in each bar). B. The formation of large, Dl− cells in intestines of flies expressing N^ICD^ in ISCs and EBs (esg::Gal4, tub::Gal80^ts^) is also rescued by over-expressing TSC1 and 2. GFP (ISC and EB), green; Dl, red; DAPI, blue. Quantification shows percentage of GFP+ cells expressing Dl (Averages and SEM; Student's Ttest; N shown in each bar).

Loss of TSC1/2 also rescued the accumulation of pros+ EE cells in N loss-of-function conditions (Figure S5D, Figure S5E), confirming a shift towards the EC fate in TSC-deficient EBs. Furthermore, co-expression of TSC1 and 2 resulted in the maintenance of small, diploid, Dl+ cells even in the presence of N^ICD^, showing that TSC2 repression is required for N-induced ISC differentiation (in both Flp-out clones and when driven by esgGal4, Figure 6). Repression of TSC1/2 function is thus a critical step in the regulation of EB differentiation.

## Discussion

### TSC function and stem cell maintenance

**Figure 7:**
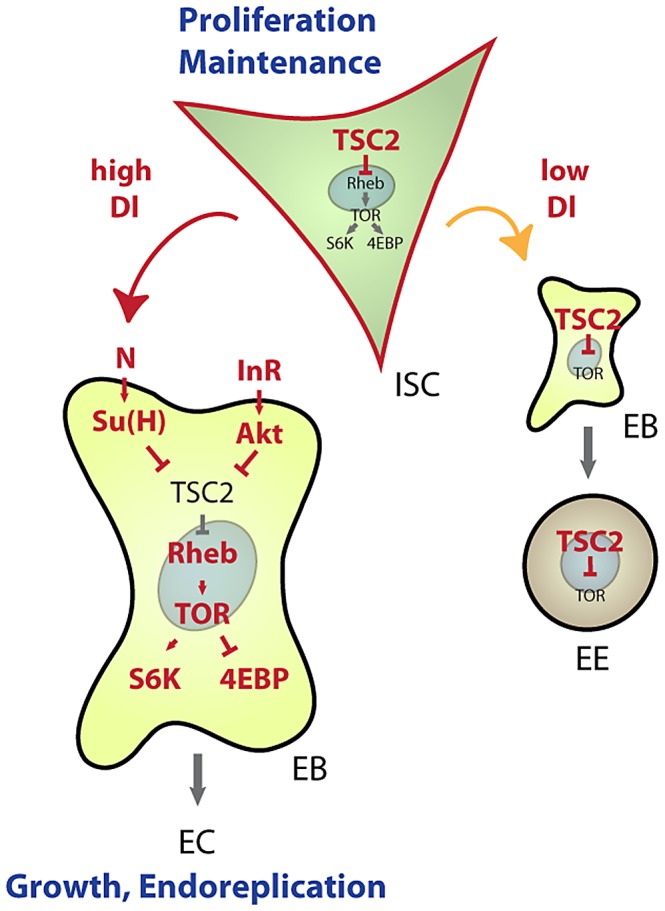
Model for TSC–mediated regulation of cell differentiation in the ISC lineage. Our results identify transcriptional repression of TSC2 as a required step in the induction of EC differentiation by the Notch signaling pathway. This regulation integrates with Insulin-mediated repression of TSC2 function to promote EC growth and endoreplication. Under conditions of low Notch activation, TSC2 expression in EBs remains high and prevents growth of cells differentiating into EE cells. Similarly, in ISCs, high TSC2 expression serves as a ‘buffer’ from nutritional changes that increase IIS activity or can otherwise activate the TOR pathway, thus promoting ISC maintenance.

Our results establish a new mechanism by which lineage commitment, differentiation and growth are coordinated in an epithelial stem cell lineage (Figure 7). This mechanism allows for the integration of nutritional signals through the IIS and TOR pathways with Notch-mediated differentiation signals: High expression of TSC2 in ISCs prevents differentiation and is thus critical for stem cell maintenance, while reducing TSC activity in EBs is required and sufficient to promote differentiation into ECs. This dynamic regulation of TSC levels in the ISC lineage intersects with the control of TSC1/2 activity by growth factor signals. Based on current models, we propose that control of TSC2 expression is required to set a threshold for the Akt-mediated inactivation of the TSC1/2 complex downstream of growth factor receptors. The TSC2 expression level would thus determine the cellular response to growth signals in the ISC lineage. Supporting this view, ISCs, which express high levels of TSC2 constitutively, do not differentiate in response to InR over-expression, but rather increase their proliferation rate. EBs, on the other hand, express less TSC2 and respond to InR activation by endoreplicating and growing into ECs.

Robust expression of TSC2 in ISCs thus prevents premature differentiation and growth of ISCs. When IIS is chronically activated (as in high nutrient conditions), however, Akt-mediated TSC1/2 complex inactivation may cause sporadic differentiation and loss of ISCs. Conversely, ISC maintenance might be improved in conditions of chronically low IIS and TOR activity. The TSC1/2 complex thus acts as a ‘buffer’ that improves ISC maintenance by isolating these cells from changing nutritional conditions. Accordingly, TSC1 deficiency leads to differentiation and loss of ISC function when flies are reared under high yeast conditions. Interestingly, these results also indicate that TOR pathway activation is not constitutive in TSC deficient ISCs, but is still inducible by nutritional changes. It is likely that amino acid-sensing signaling pathways, involving Rag GTPase complexes and the MAP4K3 and Vps34 kinases, regulate TOR in these situations [38].

An effect of TOR signaling on stem cell maintenance has previously been described in GSCs, and is consistent with recent findings that suppression of TOR activity, through rapamycin or genetic means, increases lifespan [48]–[50], [68]. While reducing IIS activity in the ISC lineage is sufficient to extend lifespan [23], additional studies will have to be performed to assess the role of TOR signaling in this context.

### Mechanisms of Notch/TSC interaction

Three pieces of evidence indicate that transcriptional repression of TSC2 occurs downstream of N activation in the ISC lineage: (i) TSC2 expression is significantly reduced in EBs with high levels of N signaling activity, (ii) forced expression of N^ICD^ suppresses TSC2 expression in ISCs, and (iii) loss of TSC2 is sufficient to rescue N loss of function phenotypes in the ISC lineage. This regulatory interaction between TSC2 and N signaling is reminiscent of recent findings in *Drosophila* sensory organ precursors, mouse embryonic fibroblasts, and mammalian cancer cells [46], [69]–[73]. However, the results reported in these studies indicated that TOR pathway activation could result in increased N cleavage and N pathway activation. In the fly SOP, activation of the TOR pathway phenocopied N gain of function phenotypes, but it was not tested whether these phenotypes were rescued in N loss of function backgrounds. It thus remained unclear whether N activation is a consequence of TOR pathway activation, or whether TOR activation is a required component of the N-induced differentiation pathway in this lineage. Our results demonstrate that in the ISC lineage, TOR activation is sufficient to drive EC differentiation even in the absence of N signaling, supporting a model in which TOR activation occurs downstream of N. In TSC mutant mouse embryonic fibroblasts, on the other hand, TOR -dependent activation of N can be observed, highlighting the close, evolutionarily conserved relationship between these two signaling pathways in the control of cell differentiation, but also suggesting that multiple, context dependent, interaction mechanisms may exist [69].

Our model implies a novel role for Su(H) as a transcriptional repressor of the *gigas* gene. This interpretation is based on the requirement of Su(H) for the N-mediated repression of the *gigas* gene both in S2 cells and in the ISC linage, as well as on the binding of Su(H) to the *gigas* promoter. A function of Su(H) as a transcriptional repressor has not previously been described, and additional studies are needed to explore its mechanism. While binding of Su(H) to the *gigas* promoter indicates a direct role in transcriptional repression of *gigas*, it is possible that Su(H) also acts indirectly to repress *gigas* expression by inducing or cooperating with transcriptional repressors. A candidate group of such repressors are encoded by Su(H) target genes, the classical Hairy and E(Spl) complex. These transcription factors are induced by Su(H) in response to Notch signaling and have been described as transcriptional repressors in other contexts [74]. Putative E(Spl) binding sites are present in the *gigas* promoter (not shown), and additional studies will therefore be of interest to dissect the requirement for individual E(Spl) complex genes in the regulation of *gigas*.

Our data further indicate that transcriptional repression of *gigas* may not be the only mechanism by which TSC2 repression is achieved in EBs. While we find that activation of N is sufficient and required for repression of TSC2 protein in EBs *in vivo*, our S2 studies indicate that the turnover rate of the TSC2 protein also has to be increased to achieve rapid reduction of TSC2 levels. It can be anticipated that the control of TSC2 ubiquitination by the cul4/ddb1/fbw5 complex may be an important regulatory mechanism here, and it will be of interest to further dissect the interaction of this complex with the N signaling pathway in the ISC lineage [44].

Characterizing these signaling interactions in ISCs in more detail is of significant interest for our understanding of somatic stem cell maintenance, proliferative homeostasis and lineage commitment. The evolutionary conservation of N and TOR signaling, as well as the similarities in the biology of *Drosophila* and vertebrate stem cell populations [7], indicate that such understanding will provide important insight into human regenerative and proliferative diseases.

## Materials and Methods

### *Drosophila* stocks and culture

The following fly stocks were obtained from the Bloomington Drosophila Stock Center: w^1118^, UAS-InR, UAS-InR^DN^, UAS-Rheb, UAS-S6K^KQ^, tub-Gal80^ts^, UAS-SuH^RNAi^ (TRiP.HM05110). UAS-TSC1^RNAi^ (Transformant ID 22252), UAS-TSC2^RNAi^ (TID 103417) and UAS-S6K^RNAi^ (TID 104369) were obtained from the Vienna *Drosophila* RNAi Center. The following lines were gifts from: Esg-Gal4, S. Hayashi; UAS-N^ICD^, UAS-Notch^RNAi^, and hsFlp; tub-Gal4, UAS-GFP; FRT82B tubGal80, N. Perrimon; Su(H)-GBE-LacZ, S. Bray; Su(H)-GBE-Gal4, S.X. Hou; UAS-TSC1, TSC2, M. Tatar; FRT40A, chico^1^, FRT82B, InR^E19^, and FRT82B, InR^353^, and *FRT40, Tor^2L1^*, *FRT40, Tor^2L19^* and *FRT40, Tor^W1251R^* by D. Drummond-Barbosa; hsFlp; FRT40A, tub-Gal80; tub-Gal4, UAS-GFP and 5966-GS, B.Ohlstein; w, hsFLP; actin, FRT, y+, FRT, Gal4, UAS::RFP, M. Uhlirova; FRT82, *Tsc1^Q87X^* and FRT82, *Rheb^2D1^*, K. Harvey.

Flies were cultured on yeast-molasses based food at 25°C with a 12 hours light/dark cycle. For TARGET experiments flies were raised at 18°C and shifted to the restrictive temperature (29°C) 3–5 days after eclosion. For clone induction (MARCM and Flp-out), 3–5 day old flies were heat shocked at 37°C for 45 minutes. For 5966GS, flies were maintained for 7 days on RU486 food (100 µl of a 5 mg/ml solution of RU486 was deposited on top of a 10 ml food vial and dried for 16 hours).

### Immunostaining and microscopy

Guts were dissected in phosphate-buffered saline (PBS) and fixed for 45 min at room temperature in 100 mM glutamic acid, 25 mM KCl, 20 mM MgSO_4_, 4 mM sodium phosphate, 1 mM MgCl_2_, and 4% formaldehyde. All subsequent washes (1 hour) and antibody incubations (4°C overnight) were performed in PBS, 0.5% bovine serum albumin and 0.1% Triton X-100.

Staining with Delta antibody was performed following the methanol-heptane fixation method described in (Lin et al., 2008).

Fluorescent *in situ* hybridization protocol was adapted from [75] using Tyramide signal amplification (TSA) and Digoxigenin (DIG) labeled RNA probes. The following primers were used to generate RNA probes for pdm1: F 5′-AGT TTG CCA AGA CCT TCA AGC AGC and R 5′-AGG GAT TGA TGC GCT TCT CCT TCT.

Primary antibodies with respective dilutions were: From Developmental Studies Hybridoma Bank: mouse anti- Armadillo and anti-Delta, 1∶100; mouse anti-Prospero, 1∶250; Cell Signaling: rabbit anti-phospho-4EBP, 1∶500; ICN: mouse anti-b-galactosidase, 1∶100; gift from Yue Xiong: rabbit anti-Gigas, 1∶500; gift from Yang Xiao-Hang: rabbit anti-Pdm1; Upstate Biotech: rabbit anti phospho histone H3, 1∶1000; Invitrogen: Alexa Fluor 568 1∶500

Confocal microscopy was performed on a Leica SP5 system. Image processing was done on NIH Image J and Adobe Photoshop.

### Cell culture

S2 cell lines stably transfected to express wild type Notch receptor (S2-Mt-N) or Delta ligand (S2-Mt-Dl) from a Cu-inducible metallothionein promoter were obtained from the Drosophila Genomic Resource Center. Both lines were cultured in M3+BPYE medium with 10% heat inactivated Fetal Calf Serum and grown under permanent selection with 0.2 µM Methotrexate (Sigma). N and Dl expression was induced separately with 600 mM CuSO_4_ for 24 hours and the two cell lines were then co-cultured in 1∶1 ratio for the indicated times.

For RNAi experiments, double-stranded RNAs were synthesized against GFP and Suppressor of Hairless using T7 promoters (Ambion MEGAscript RNAi kit). S2-Mt-N and S2-Mt-Dl cells were cultured separately and only S-Mt-N cells were treated with GFP dsRNA (control) or with SuH dsRNA for 3 days. After two days of dsRNA treatment both cell lines were induced with 600 mM CuSO_4_ for 24 hours. After three days of dsRNA treatment, S2-Mt-N cells and S2-Mt-Dl cells were co-cultured at a 1∶1 ratio for the indicated times. Knockdown of Su(H) was confirmed by RT-PCR (not shown).

### Chromatin immunoprecipitation (ChIP)

∼1×10^7^ cells were collected from triplicate cell cultures of S2-Mt-N cells (control) or of 2 h co-cultures of S2-Mt-N and S2-Mt-Dl. Cells were cross-linked using ∼1.1% formaldehyde and ChIP was performed using the abcam ChIP kit (ab500). Cells were sonicated on ice using a Branson Sonicator (power 4, 50×10 second pulses with 30 second intervals; average size of genomic DNA fragments was ∼500 bp). Sheared chromatin was incubated with 5 µg of rabbit anti-GFP (invitrogen; negative control) and goat anti-SuH (Santa Cruz Biotechnology) for 24 hours and then precipitated using Protein G Sepharose (Fast Flow; Sigma). De-crosslinking and DNA purification was performed according to kit instructions (ab500). DNA from different ChIP samples was analyzed for enrichment using real time PCR using the following primer sets: gig 1: 5′-ACAAACGCAAAGTTGGCGAC-3′ and 5′-GTGTGCAACCAGTAATTCCTAGCC-3′; gig 2: 5′-AAGTTGTTCCTCAAATCGCTGCCG-3′ and 5′-ATTGAAGTTGTGCAGCTGCGTGTC-3′; actin5C: 5′-ATTCAACACACCAGCGCTCTCCTT-3′ and 5′-ACCGCACGGTTTGAAAGGAATGAC-3′.

### Reverse transcription and real-time PCR

Total RNA was extracted using Trizol. cDNAs were synthesized using oligo-dT primers and real-time RTPCR was performed on a BioRad iQ5 detection system (using SYBR Green and ΔΔCt quantification method). Gigas and Suppressor of Hairless expression levels were quantified relative to Actin5c expression.

### Western blot

Cell samples were resolved using 5% (for N^ICD^) or 10% (for tubulin) SDS-polyacrylamide gel electrophoresis, transferred to nitrocellulose membranes using semi-dry transfer, and probed with the following primary antibodies: mouse anti-N^ICD^ (DSHB, 1∶10,000), mouse anti-alpha-tubulin (Sigma, 1∶5000). Antibodies were detected using horseradish peroxidase-conjugated secondary antibodies and the ECL detection system (Amersham).

## Supporting Information

Figure S1 Relates to Figure 1. Tor/IIS interaction. A. Inhibition of TOR Pathway (escargotGal4-mediated over-expression of TSC1/2 and S6KRNAi in ISCs and EBs) in Insulin gain-of-function background rescues the InR growth phenotype (large nuclei and cell size). B. Quantification of mitoses per gut (# of pH3+ cells) and nuclear size. TOR pathway inactivation rescues InR-mediated increase in nuclear size but it has no effect on InR-mediated increase in the number of mitotic stem cells. C. Controls to test the emergence of spontaneous clones in non-heat shocked animals. Non-heat shocked controls (nhc) show significantly fewer GFP-labeled clones than heat-shocked animals, both at 1 day and 5 days after the heat shock. Blue: DAPI, Green: GFP.
(TIF)

Figure S2 Relates to Figure 2. Sporadic growth of TSC mutant ISCs. A. Examples for the guts used for scoring clones sizes in Figure 2A. B. Many TSC1 mutant clones contain small Dl+ ISCs (white arrowheads), while some clones contain large, polyploid Dl+ cells (red arrowheads). GFP (MARCM clones), green; Delta, red; DAPI, blue. C. At all analyzed ages, TSC2 mutant clones (using the *gig^192^* mutant allele) can be detected that contain small Dl+ ISCs (white arrowheads), but also clones that contain large, polyploid Dl+ cells (red arrowheads). GFP (MARCM clones), green; Delta, red; DAPI, blue. D. Examples of MARCM clones expressing TSC2^RNAi^. Note that, as with *Tsc1^Q87X^*, polyploid Dl+ cells are observed in some clones, while others retain diploid Dl+ ISCs (arrowheads point to selected Dl+ cells; white-small; red-large).
(TIF)

Figure S3 Relates to Figure 3. A. TSC2 mutant clones (*gig^192^*) have elevated levels of p4EBP. GFP (MARCM clones, 1 day after heat shock), green; p4EBP, red; DAPI, blue. B. Over-expression of TSC1 and 2, or S6K^RNAi^ in EBs and ECs impairs endoreplication. GFP+/Dl+ cells in flies expressing GFP, TSC1+2, or S6K^RNAi^ in ECs and EBs under the control of *5966::GS* maintain low DNA content compared to EC. Flies were exposed to RU486 for 7 days. DNA content was analyzed by integrating DAPI intensity values across all pixels for individual nuclei using TCSNT software from Leica (arbitrary units [AU] are listed). Values are shown for ECs (GFP+/Dl−), misdifferentiated EBs (GFP+/Dl+), and ISCs (GFP−/Dl+). Bars represent averages and standard deviations (N = 5–10 nuclei, from 3 independent guts), Student's Ttest. C. Quantification of EE cells found in midguts of flies expressing TSC1+2 in EBs. Number of EEs (pros+cells) detected was normalized to the number of EBs (GFP+) cells in the gut to control for proliferative activity of ISCs. Averages and Standard Deviation, Student's Ttest.
(TIF)

Figure S4 Related to Figure 4. Western blot against N^ICD^ confirming N activation in S2 cell co-cultures. Anti-alpha Tubulin was used for loading control.
(TIF)

Figure S5 Relates to Figure 5. Loss of TSC1/2 promotes EC differentiation and reduces EE cell formation in N loss of function backgrounds. A. Intestines expressing N^RNAi^ and TSC1^RNAi^ or TSC2^RNAi^ under the control of esg::G4, tub::Gal80^ts^, UAS::GFP for 7 days. Loss of TSC1 expression in N^RNAi^-expressing ISCs and EBs results in GFP+ cells that display a Phalloidin+brush border. Expression of TSC2^RNAi^ results in larger, polyploidy cells (see also B), but these cells do not display a brush border. Phall, red; DAPI, blue. B. Loss of TSC1 can restore differentiation markers in N^RNAi^ loss-of-function background. Intestines expressing N^RNAi^ and TSC1 or 2^RNAi^ under the control of esg::G4, tub::Gal80^ts^ for 7 days. *In situ* hybridization for *pdm1* reveals that cells expressing N^RNAi^ alone have decreased *pdm1* expression compared to surrounding ECs, while cells co-expressing TSC1^RNAi^ and N^RNAi^ (GFP+) have similar levels of *pdm1* mRNA expression compared to wild-type ECs. Consistent with the observed lack of brush border, expression of TSC2^RNAi^, however, is not sufficient to restore *pdm1* expression. pdm1 RNA red; DAPI, blue. C. Loss of TSC1 or TSC2 induces polyploidy in N^RNAi^ loss-of-function background. N and TSC1 or TSC2 loss of function induced in ISCs and EBs by expressing N^RNAi^, TSC1^RNAi^, and/or TSC2^RNAi^ under the control of esg::G4, tub::G80^ts^. DNA content was analyzed as in Figure 5C. GFP− ECs, pros+ EEs, and GFP+ ISC/EBs were measured. Bar represent averages and standard deviations (N = 5–10 nuclei, from 3 independent guts), Student's Ttest. D. Co-expression of TSC1^RNAi^ or TSC2^RNAi^ is sufficient to rescue the increase in EE cells observed in intestines expressing N^RNAi^ under the control of esg::G4, tub::G80^ts^. The number of pros+cells as a fraction of all GFP+ cells is shown in the graph. Averages and SEM from n = 5–10 guts, Student's Ttest. E. Fraction of MARCM clones expressing N^RNAi^ or N^RNAi^ in *Tsc1^q87X^* background that contain EE cells is shown (N = 32 for N^RNAi^ and N = 15 for N^RNAi^, *Tsc1^q87X^*).
(TIF)
