# Supplementary figures and images for: Notch-Mediated Suppression of TSC2 Expression Regulates Cell Differentiation in the Drosophila Intestinal Stem Cell Lineage
Source: PLoS Genet. 2012 Nov 8;8(11):e1003045. doi: 10.1371/journal.pgen.1003045 (PMC3493453; doi:10.1371/journal.pgen.1003045)

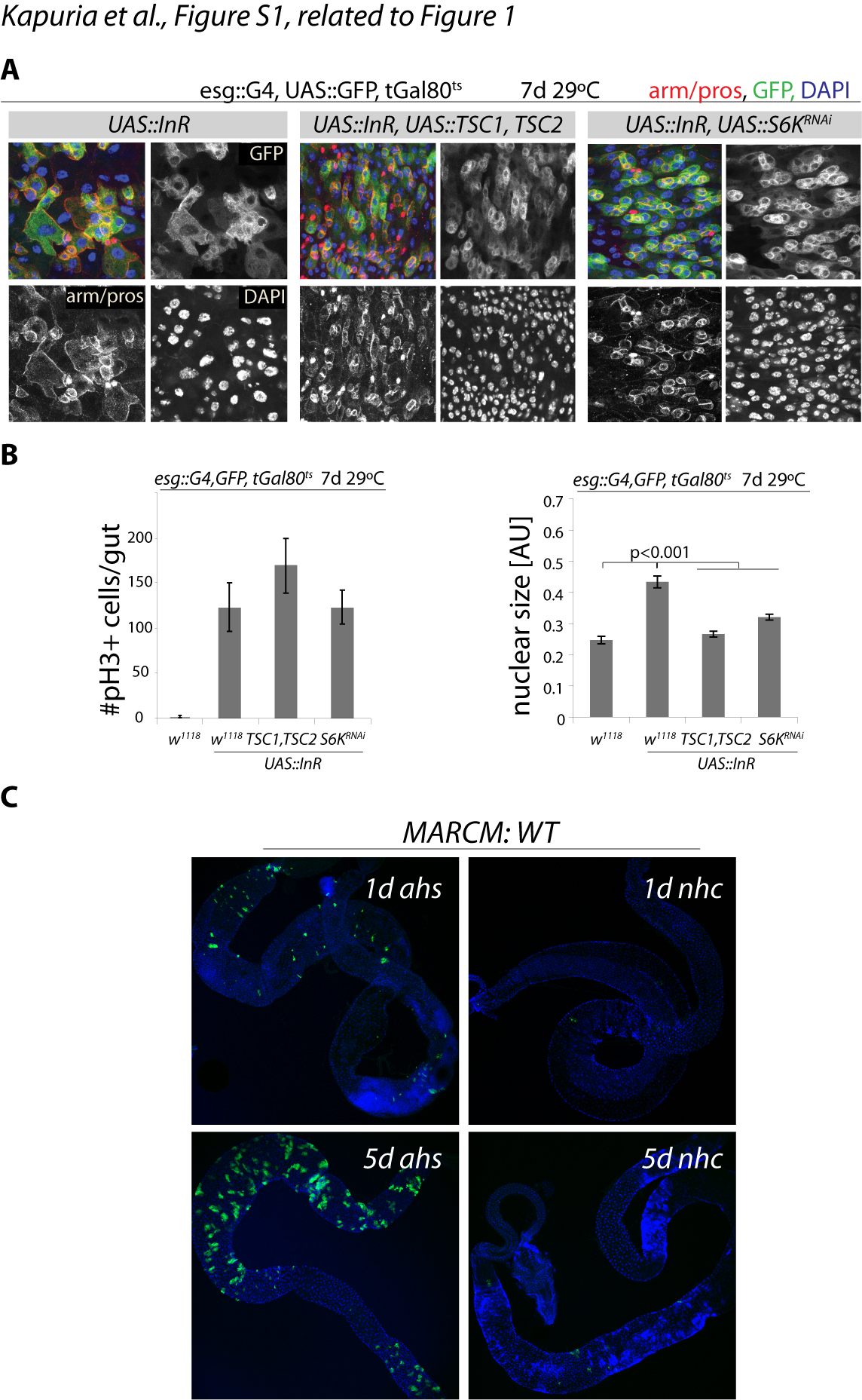

Supplement: Figure S1 — Relates to Figure 1. Tor/IIS interaction. A. Inhibition of TOR Pathway (escargotGal4-mediated over-expression of TSC1/2 and S6KRNAi in ISCs and EBs) in Insulin gain-of-function background rescues the InR growth phenotype (large nuclei and cell size). B. Quantification of mitoses per gut (# of pH3+ cells) and nuclear size. TOR pathway inactivation rescues InR-mediated increase in nuclear size but it has no effect on InR-mediated increase in the number of mitotic stem cells. C. Controls to test the emergence of spontaneous clones in non-heat shocked animals. Non-heat shocked controls (nhc) show significantly fewer GFP-labeled clones than heat-shocked animals, both at 1 day and 5 days after the heat shock. Blue: DAPI, Green: GFP. (TIF) [file pgen.1003045.s001.tif]

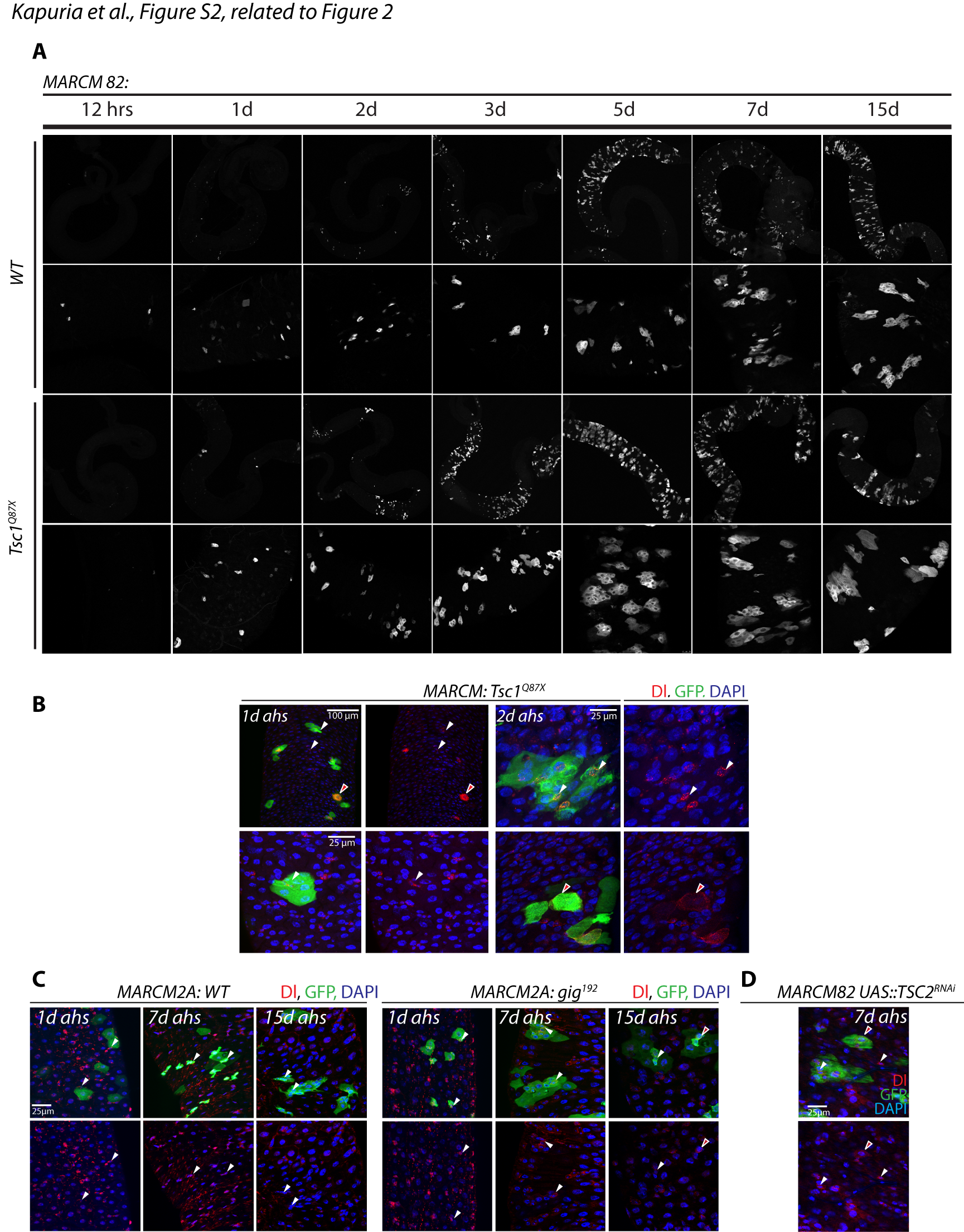

Supplement: Figure S2 — Relates to Figure 2. Sporadic growth of TSC mutant ISCs. A. Examples for the guts used for scoring clones sizes in Figure 2A. B. Many TSC1 mutant clones contain small Dl+ ISCs (white arrowheads), while some clones contain large, polyploid Dl+ cells (red arrowheads). GFP (MARCM clones), green; Delta, red; DAPI, blue. C. At all analyzed ages, TSC2 mutant clones (using the gig192 mutant allele) can be detected that contain small Dl+ ISCs (white arrowheads), but also clones that contain large, polyploid Dl+ cells (red arrowheads). GFP (MARCM clones), green; Delta, red; DAPI, blue. D. Examples of MARCM clones expressing TSC2RNAi. Note that, as with Tsc1Q87X, polyploid Dl+ cells are observed in some clones, while others retain diploid Dl+ ISCs (arrowheads point to selected Dl+ cells; white-small; red-large). (TIF) [file pgen.1003045.s002.tif]

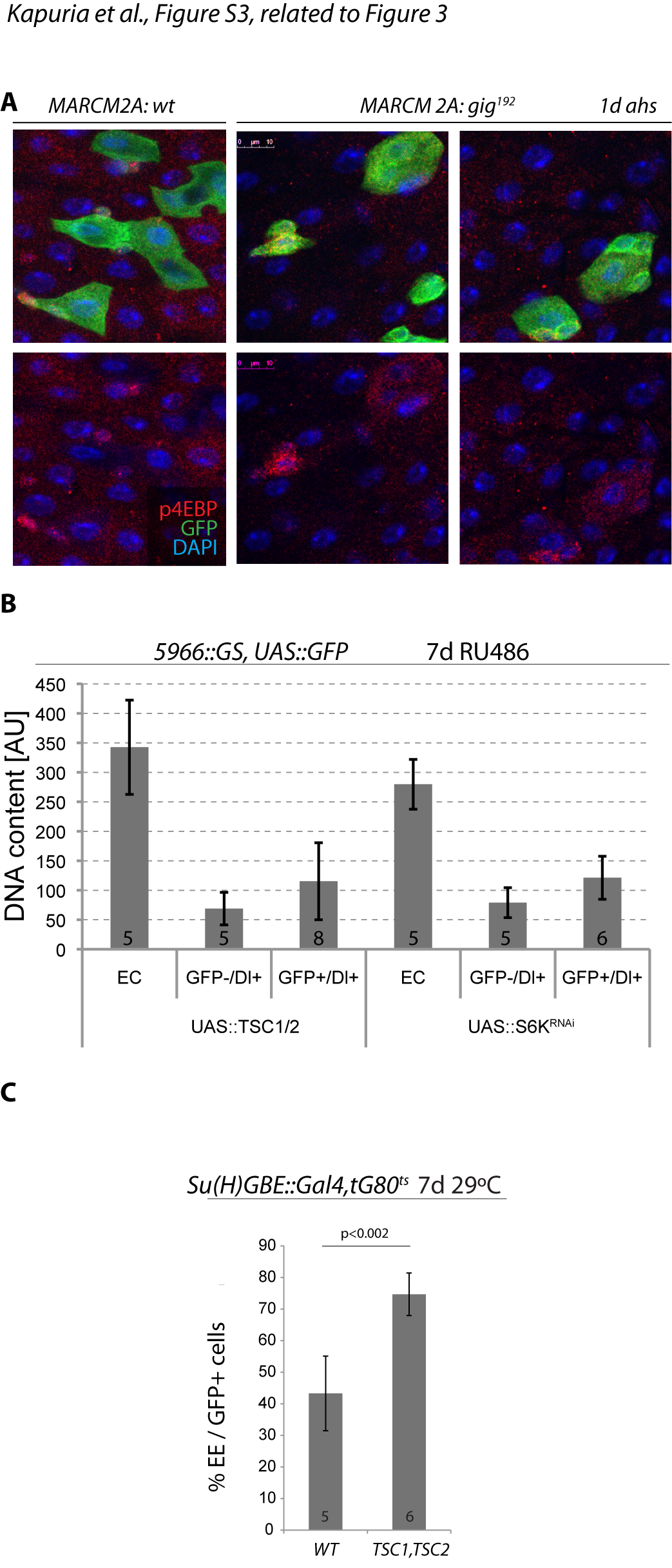

Supplement: Figure S3 — Relates to Figure 3. A. TSC2 mutant clones (gig192) have elevated levels of p4EBP. GFP (MARCM clones, 1 day after heat shock), green; p4EBP, red; DAPI, blue. B. Over-expression of TSC1 and 2, or S6KRNAi in EBs and ECs impairs endoreplication. GFP+/Dl+ cells in flies expressing GFP, TSC1+2, or S6KRNAi in ECs and EBs under the control of 5966::GS maintain low DNA content compared to EC. Flies were exposed to RU486 for 7 days. DNA content was analyzed by integrating DAPI intensity values across all pixels for individual nuclei using TCSNT software from Leica (arbitrary units [AU] are listed). Values are shown for ECs (GFP+/Dl−), misdifferentiated EBs (GFP+/Dl+), and ISCs (GFP−/Dl+). Bars represent averages and standard deviations (N = 5–10 nuclei, from 3 independent guts), Student's Ttest. C. Quantification of EE cells found in midguts of flies expressing TSC1+2 in EBs. Number of EEs (pros+cells) detected was normalized to the number of EBs (GFP+) cells in the gut to control for proliferative activity of ISCs. Averages and Standard Deviation, Student's Ttest. (TIF) [file pgen.1003045.s003.tif]

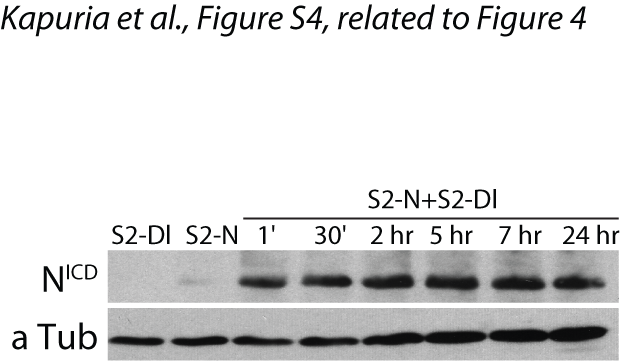

Supplement: Figure S4 — Related to Figure 4. Western blot against NICD confirming N activation in S2 cell co-cultures. Anti-alpha Tubulin was used for loading control. (TIF) [file pgen.1003045.s004.tif]

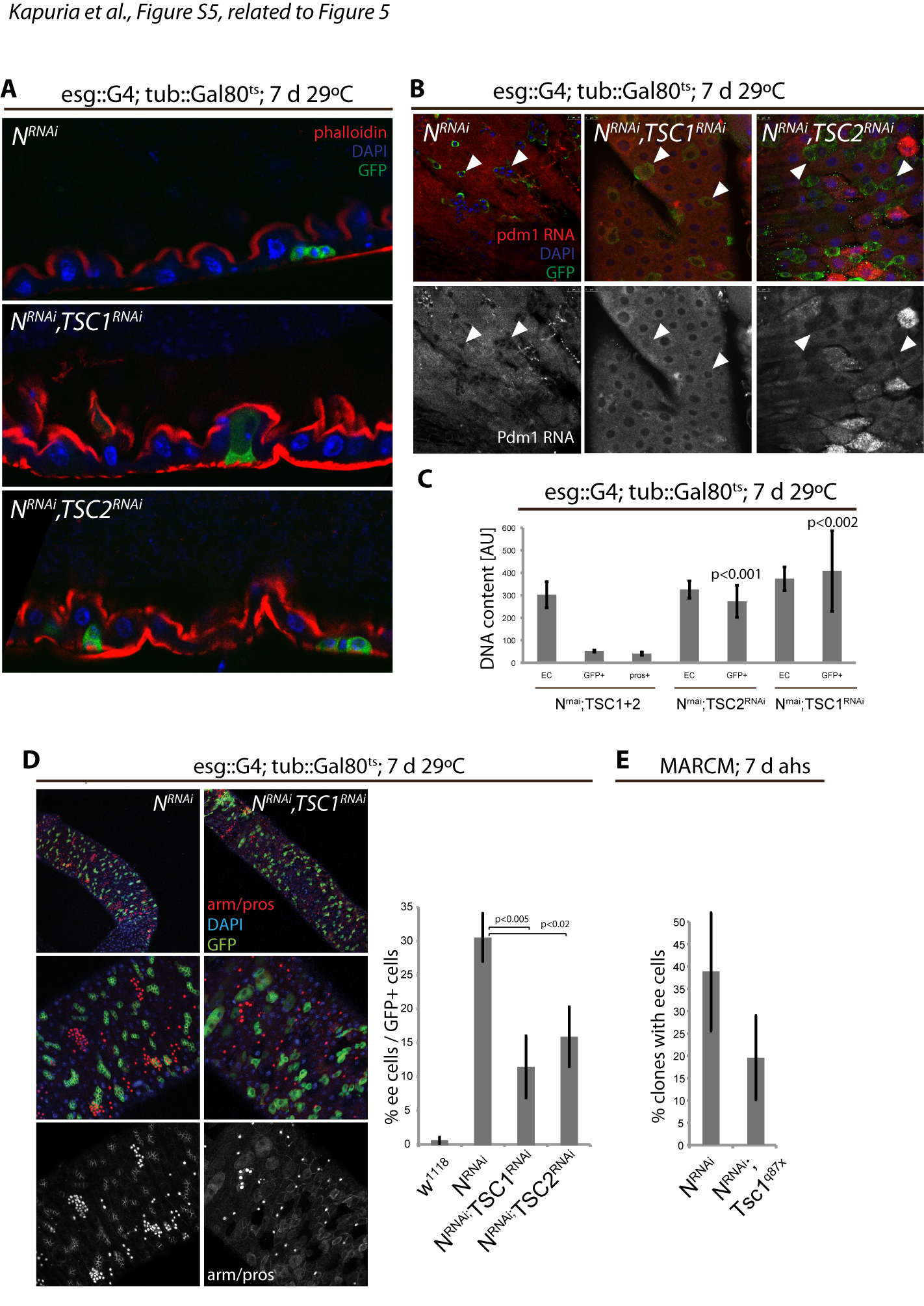

Supplement: Figure S5 — Relates to Figure 5. Loss of TSC1/2 promotes EC differentiation and reduces EE cell formation in N loss of function backgrounds. A. Intestines expressing NRNAi and TSC1RNAi or TSC2RNAi under the control of esg::G4, tub::Gal80ts, UAS::GFP for 7 days. Loss of TSC1 expression in NRNAi-expressing ISCs and EBs results in GFP+ cells that display a Phalloidin+brush border. Expression of TSC2RNAi results in larger, polyploidy cells (see also B), but these cells do not display a brush border. Phall, red; DAPI, blue. B. Loss of TSC1 can restore differentiation markers in NRNAi loss-of-function background. Intestines expressing NRNAi and TSC1 or 2RNAi under the control of esg::G4, tub::Gal80ts for 7 days. In situ hybridization for pdm1 reveals that cells expressing NRNAi alone have decreased pdm1 expression compared to surrounding ECs, while cells co-expressing TSC1RNAi and NRNAi (GFP+) have similar levels of pdm1 mRNA expression compared to wild-type ECs. Consistent with the observed lack of brush border, expression of TSC2RNAi, however, is not sufficient to restore pdm1 expression. pdm1 RNA red; DAPI, blue. C. Loss of TSC1 or TSC2 induces polyploidy in NRNAi loss-of-function background. N and TSC1 or TSC2 loss of function induced in ISCs and EBs by expressing NRNAi, TSC1RNAi, and/or TSC2RNAi under the control of esg::G4, tub::G80ts. DNA content was analyzed as in Figure 5C. GFP− ECs, pros+ EEs, and GFP+ ISC/EBs were measured. Bar represent averages and standard deviations (N = 5–10 nuclei, from 3 independent guts), Student's Ttest. D. Co-expression of TSC1RNAi or TSC2RNAi is sufficient to rescue the increase in EE cells observed in intestines expressing NRNAi under the control of esg::G4, tub::G80ts. The number of pros+cells as a fraction of all GFP+ cells is shown in the graph. Averages and SEM from n = 5–10 guts, Student's Ttest. E. Fraction of MARCM clones expressing NRNAi or NRNAi in Tsc1q87X background that contain EE cells is shown (N = 32 for NRNAi and N = 15 for NRNA [file pgen.1003045.s005.tif]
